# Morphological analysis of the omovertebral bone and scapula in children with sprengel deformity using three-dimensional computed tomography

**DOI:** 10.3389/fped.2025.1730643

**Published:** 2025-12-15

**Authors:** Tao Li, Jiajing Meng, Yinghan Lei, Hai Jiang

**Affiliations:** 1 Northwest Women’s and Children’s Hospital, Xi'an, China; 2 Pediatric Orthopedics Department, Xian, China; 3 The Third Affiliated Hospital of Xi'an Medical University, Xi'an, China; 4 Medical Imaging Department, Xi'an, China

**Keywords:** children, sprengel's deformity, omovertebral bone, morphology, three-dimensional

## Abstract

**Objective:**

Sprengel deformity represents a rare complex congenital anomaly in children, characterized by malposition and dysplasia of the scapula. The omovertebral bone is considered to be the key pathoanatomic structure that affects the scapula and prevents its normal descent. Three-dimensional computed tomography (3D-CT) was employed in this study to clarify the morphological abnormalities of both the omovertebral bone and the scapula, thereby enhancing our understanding of this uncommon condition.

**Methods:**

From January 2,012 to June 2024, we enrolled 12 children (6 males and 6 females; mean age 4 years, range 2–7) with Sprengel deformity, confirmed by imaging to possess an omovertebral bone. Preoperative CT scans with 3D reconstruction were performed on all patients. We assessed the spatial location and morphology of the omovertebral bone in each case and quantified the following parameters: the height-to-width ratio of both scapulae, the extent of superior displacement of the affected scapula, and its 3D-CT rotational divergence (angle of tilt) compared to the contralateral side.

**Results:**

Based on the Cavendish grading system, 2 shoulders were classified as grade 2, and 10 were grade 3. According to the Rigault classification, 7 shoulders were grade 2, and 5 were grade 3. The mean height-to-width ratio of the affected scapulae was significantly reduced compared to the contralateral side. The infraspinous portion of the vertebral border exhibited convexity in 11 out of 12 cases, and all affected scapulae demonstrated rotational deformity. The omovertebral bone is most frequently connected to the cervical spine at C6. Its scapular attachment site was infraspinous along the vertebral border in 9 children and mesoscapular in 3. The omovertebral bones displayed diverse morphologies, predominantly irregular in shape.

**Conclusion:**

By precisely delineating the diverse morphology and spatial location of the omovertebral bone and the complex scapular distortion in three dimensions, 3D-CT provides invaluable pathoanatomic data that significantly advance our understanding of Sprengel deformity in children.

**Level of Evidence:**

Level IV—observational study design.

## Introduction

Sprengel deformity is a rare congenital condition of the shoulder girdle, characterized fundamentally by the failure of the embryonic scapula to complete its normal descent from the cervical region to its anatomical location on the posterior thoracic wall. This results in a small, elevated, and rotated scapula, which leads to significant cosmetic concerns and functional limitations in shoulder abduction and movement (1–3).

The treatment for Sprengel deformity is primarily surgical, indicated for significant functional impairment or cosmetic deformity. The main goals of surgery are to improve shoulder function, particularly abduction, and to enhance the cosmetic appearance by lowering the scapula. Several surgical techniques have been developed, with the modified Woodward procedure being the most widely accepted (4–8). This technique involves the release and resection of the omovertebral connection, fibrous bands, and tight musculotendinous attachments, followed by the reattachment of muscles at a lower position. For older children, an additional step of excising the prominent superomedial portion of the scapula (a procedure known as scapuloplasty) is often performed to improve contour and prevent secondary winging.

The efficacy of treatment and long-term prognosis are highly dependent on the severity of the deformity, the presence of an omovertebral bone, the degree of muscle hypoplasia, and the age of the patient at the time of intervention. Surgery generally yields satisfactory outcomes, with significant improvements in both cosmetic appearance and shoulder abduction (9–11). However, complete normalization of function and appearance is rarely achieved. Potential complications encompass brachial plexus palsy from excessive traction during scapular repositioning, keloid formation, and deformity recurrence. Early surgical intervention, optimally undertaken between ages 3 and 6, is linked to superior functional and cosmetic outcomes, capitalizing on the enhanced plasticity and remodeling potential of juvenile tissues.

This investigation characterizes the morphology of the omovertebral bone and the associated scapular anomaly in Sprengel deformity, with the goal of correlating these features with clinical findings and providing an anatomical basis for established surgical approaches.

## Methods

Between January 2012 and June 2024, 30 children with Sprengel deformity were identified across two clinical centers. Among them, 12 children (6 boys and 6 girls; mean age 4 years, range 2–7) who met the following criteria were enrolled: (1) presence of an omovertebral bone, and (2) availability of complete preoperative imaging data, particularly three-dimensional CT. Patients were excluded for (1) absence of an omovertebral bone, (2) bilateral omovertebral bones, or (3) incomplete imaging data. All patients underwent preoperative CT with 3D reconstruction. We assessed the spatial location and morphology of the omovertebral bone in each case and measured the following parameters: the height-to-width ratio of both scapulae, the degree of superior displacement of the affected scapula, and its 3D-CT rotational divergence (angle of tilt) compared to the contralateral side.

According to Cavendish classification (12), 2 cases were classified as grade 2, 10 cases as grade 3. According to Rigault's classification (13), 7 cases were classified as grade 2, 5 cases as grade 3. Preoperative CT scans with 3D-CT reconstruction were performed to assess the morphological changes of the omovertebral bone and scapula.

### Measurement technique

**Figure 1:**
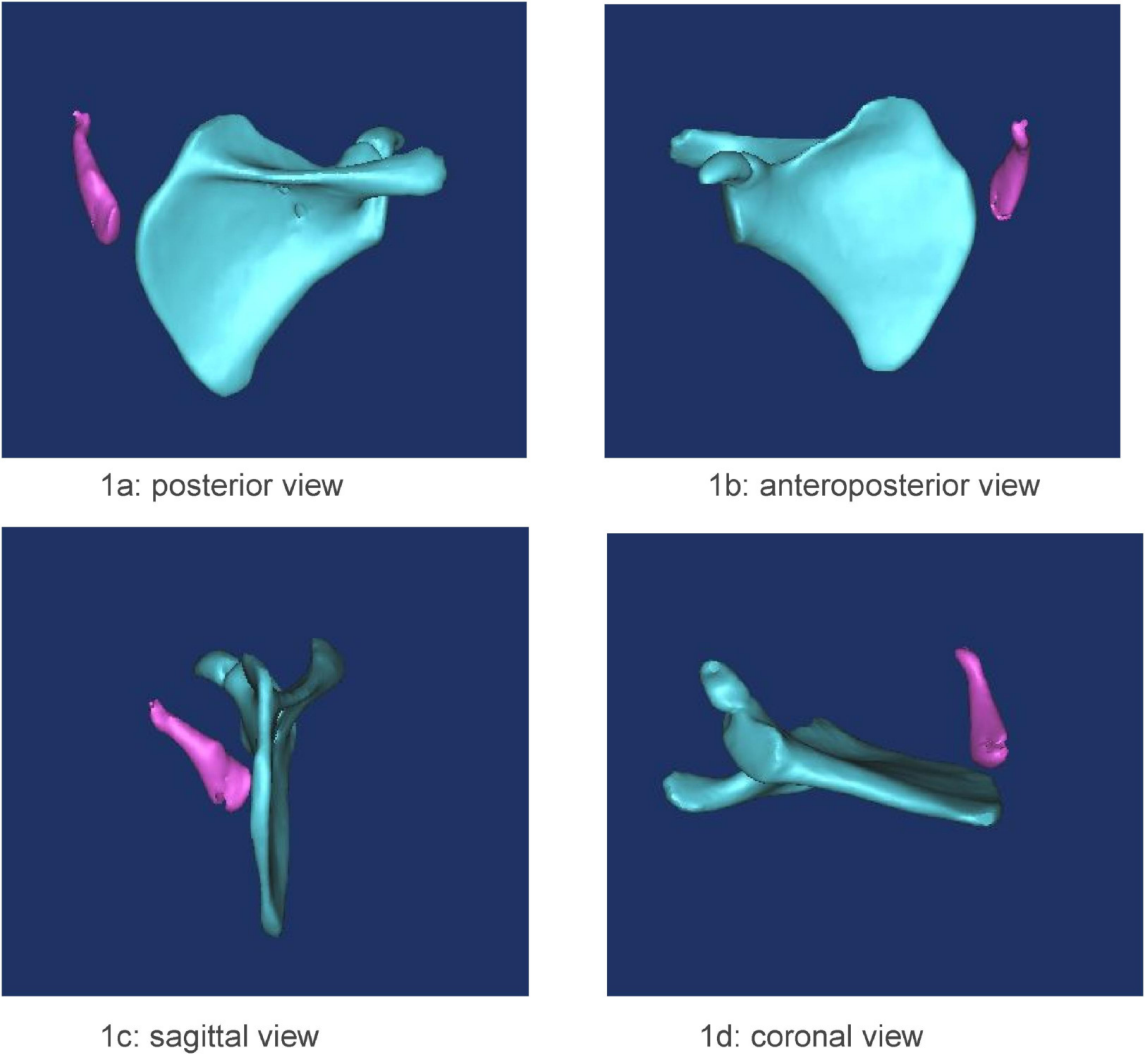
**(a–d)** case1. The relationship of the omovertebral bone and the scapula was observed from four views. The four views demonstrate the spatial alignment of the omovertebral bone, extending from the medial border of the scapula along the anterolateral axis to the inferior border.

**Figure 2:**
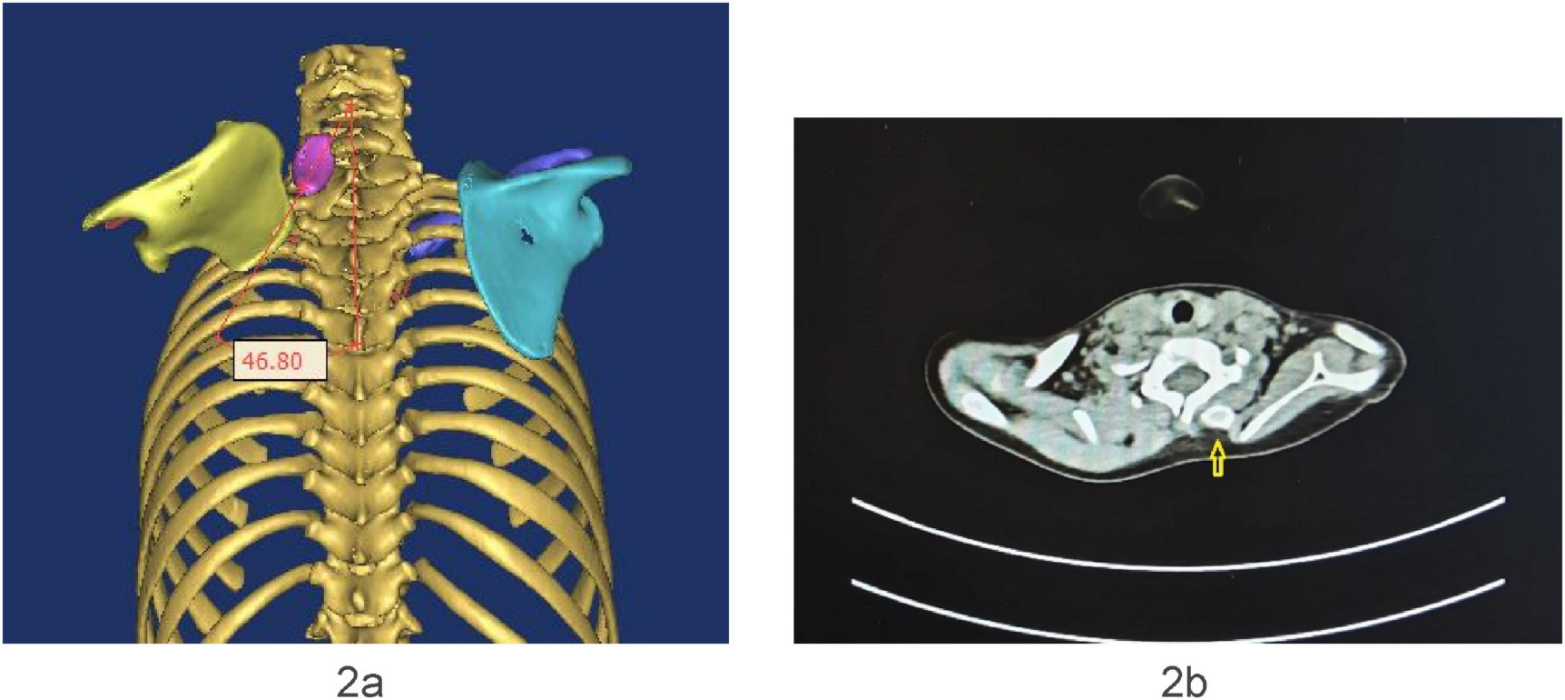
**(a,b)** Case8. The angle between the longitudinal axis of the omovertebral bone and the spinal axis was 46.80°. The omovertebral bone was indicated by the yellow arrow.

The omovertebral bone was assessed from multiple views, including posterior, anteroposterior, sagittal, and coronal (Figures 1a,d). The angle between the longitudinal axis of the omovertebral bone and the spinal axis was also measured (Figures 2a,b).

**Figure 3:**
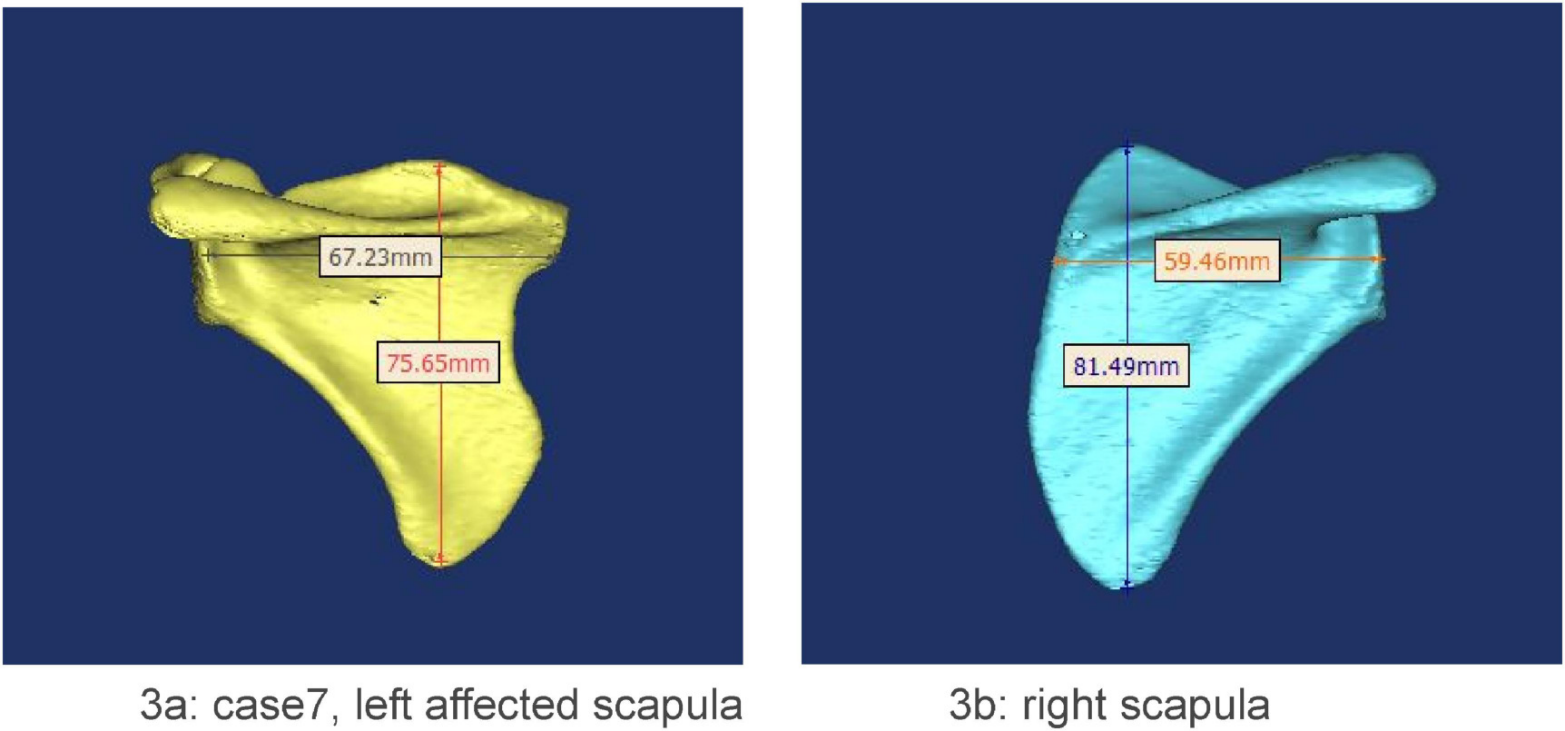
**(a,b)** the techniques for measuring scapular height and width were demonstrated.

Scapular height was defined as the distance from the superior angle to the inferior angle, measured parallel to the glenoid fossa. Scapular width was measured from the glenoid fossa to the most medial point of the vertebral border, perpendicular to the glenoid (14) (Figures 3a,b).

**Figure 4:**
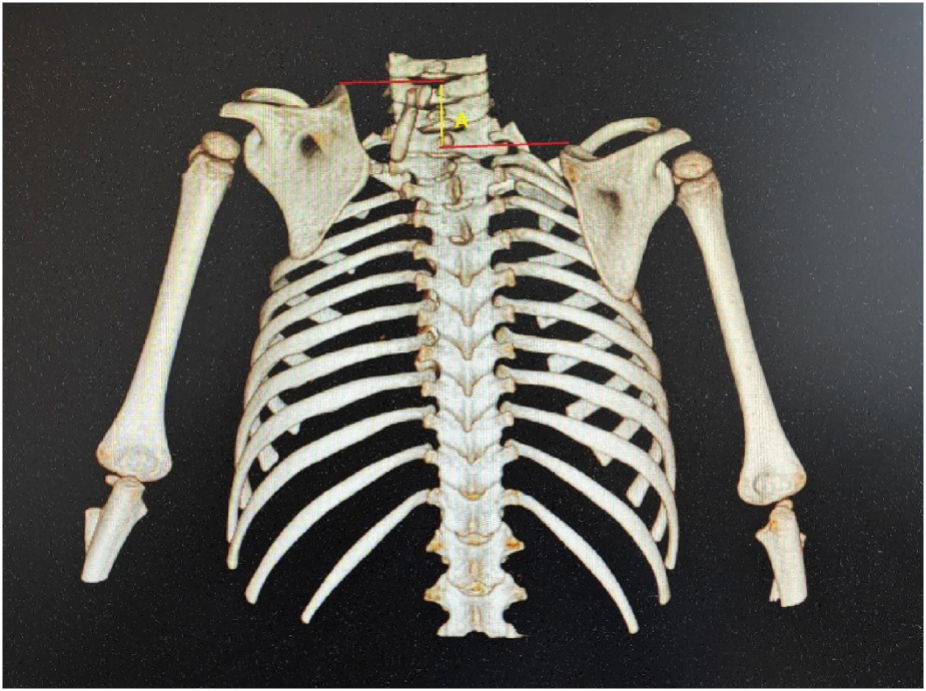
Case 5. The superior displacement **(A)** of the affected scapula was the distance between the two lines.

**Figure 5:**
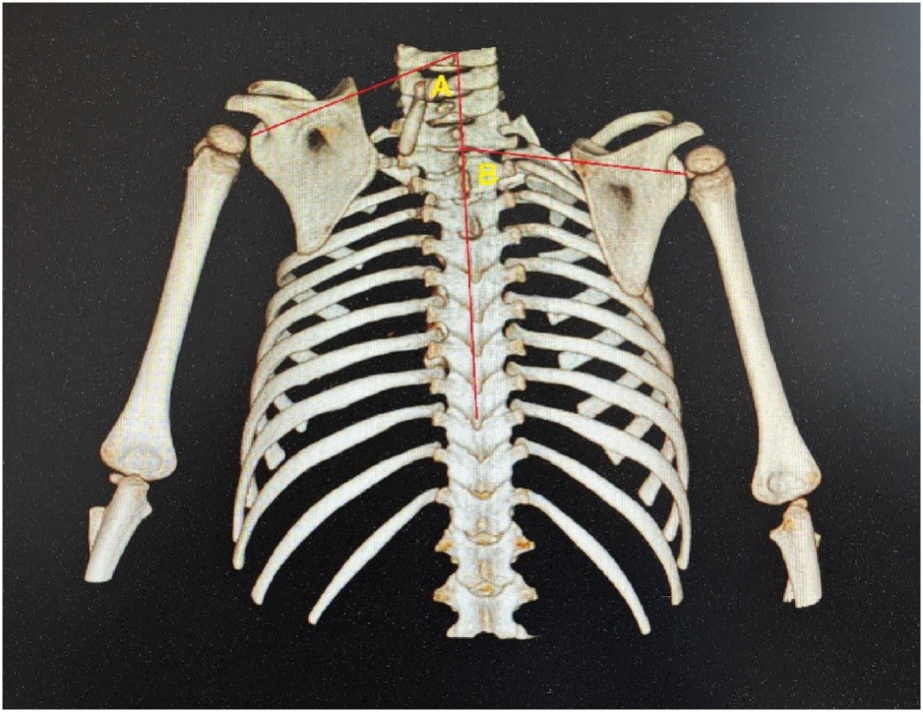
Case 5. The techniques for measuring scapular rotational angles were demonstrated. Rotational deformity = B-A.

The height-to-width ratio of the scapula was then calculated from the posterior view. Ratio = height/ width. Superior displacement of the affected scapulae was measured relative to the contralateral side (Figure 4). Lines were drawn from the tip of the superior angle perpendicular to the vertebral axis line. Rotational differences were assessed by comparing the tilt angles between the affected and contralateral sides (Figure 5). A line was drawn from the center of the glenoid cavity along the medial border of the mesoscapula, and another line was drawn parallel to the vertebral axis. The angle formed between these two lines defines the scapular rotation.

An independent assessment of the data was performed by a pediatric orthopedic surgeon (Dr. JH) and a radiologist (Dr. M JJ). To evaluate intra-observer agreement, both investigators repeated the measurements following a two-week washout period. For the final analysis, the average of all measurements from both observers was used. Intra- and interobserver reliability were assessed using the intraclass correlation coefficient (ICC). The ICC for intraobserver reliability was 0.91, and for interobserver reliability was 0.89, indicating excellent consistency.

Data were presented as mean ± standard deviation. A paired t-test was used to compare the contralateral side with the affected side. A *P* value of less than 0.05 was considered to be statistically significant.

## Results

**Figure 6:**
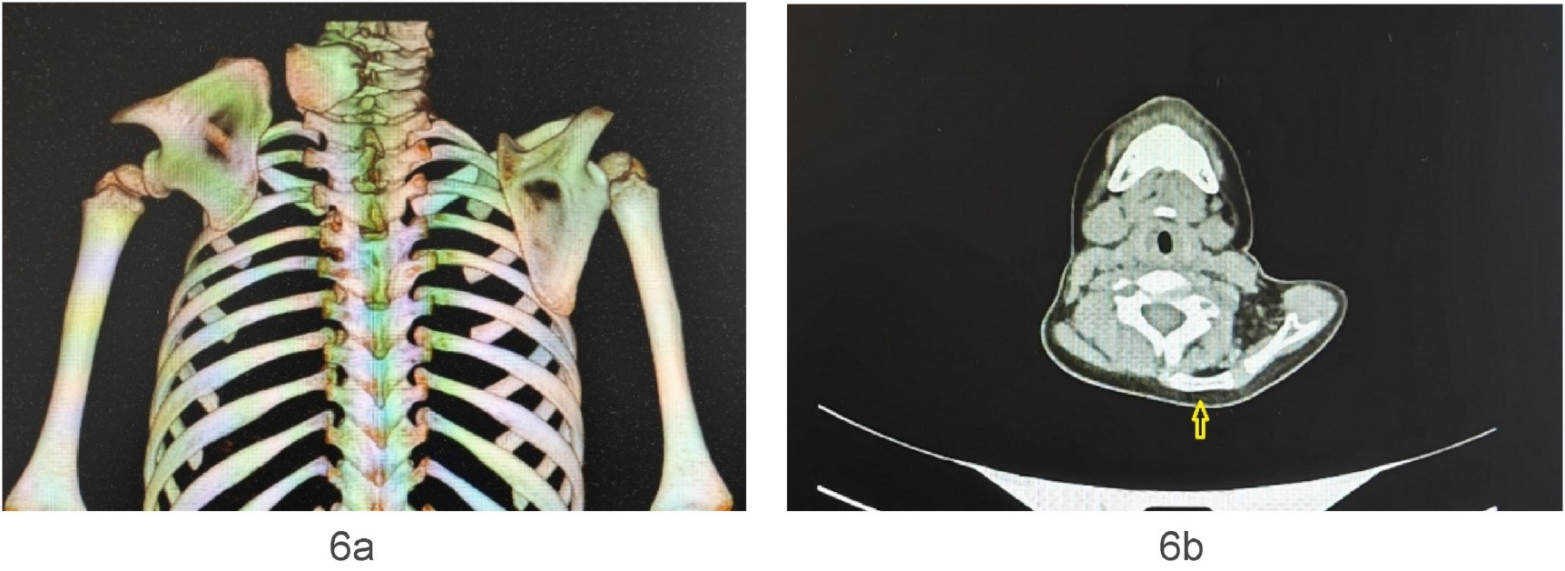
**(a, b)** case 10. The tethering point to the scapula was in the infraspinous portion, irregular in shape. The yellow arrow identified the omovertebral bone.

**Figure 7:**
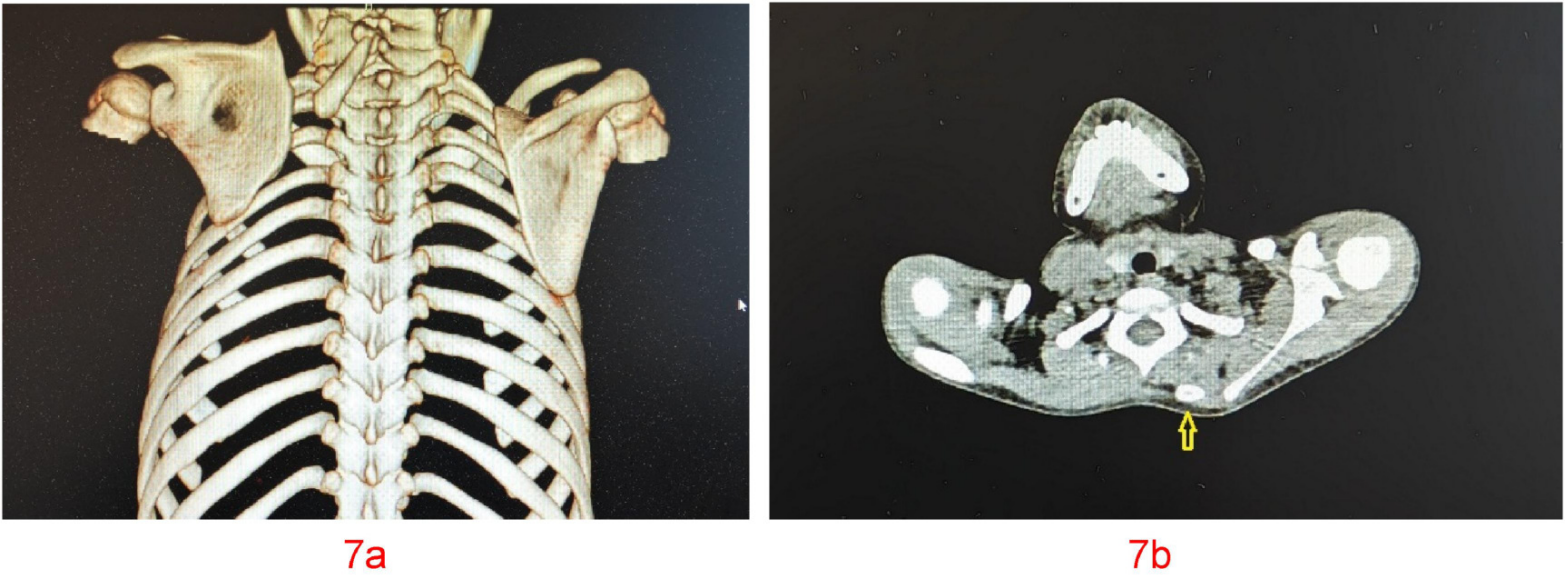
**(a, b)** case 9. The shape of the omovertebral bone was cylindrical. The yellow arrow pointed to the omovertebral bone.

The omovertebral bone most frequently attached to the cervical spine at C6 (5 cases), followed by C7 (2 cases); the remaining 5 cases were at other levels with 2 or 3 cervical sections. In the majority of the shoulders, the omovertebral bone was tethered to the convexity of the infraspinous portion of the vertebral border, whereas in three shoulders, it anchored to the convexity of the mesoscapular portion. The morphology of the omovertebral bone was diverse. Most of the shapes were irregular (Figures 6a,b). Some exhibited rhomboid or cylindrical shapes (Figures 7a,7b).

**Figure 8:**
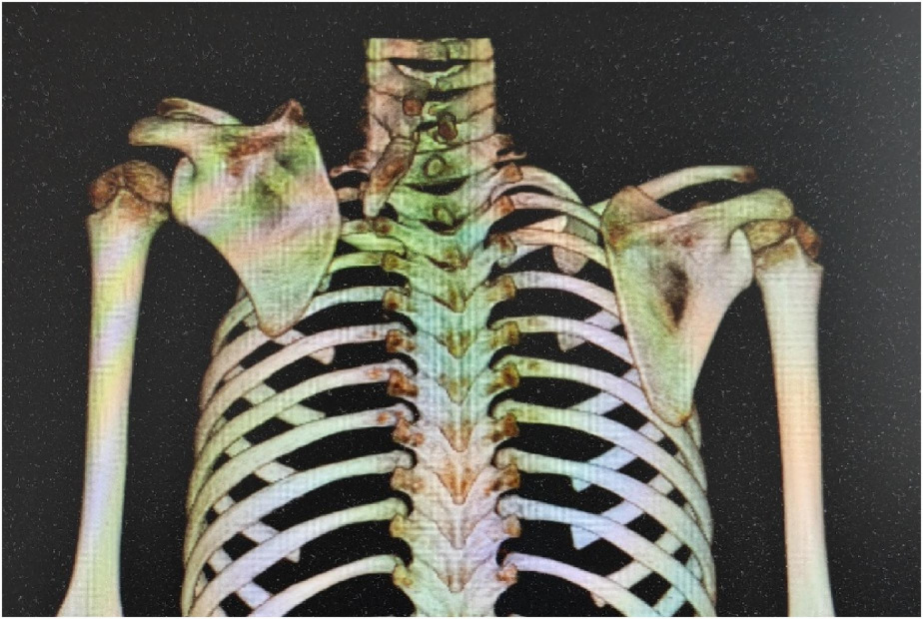
Case 12. A cartilaginous connection was observed at the scapular attachment site of the omovertebral bone.

**Figure 9:**
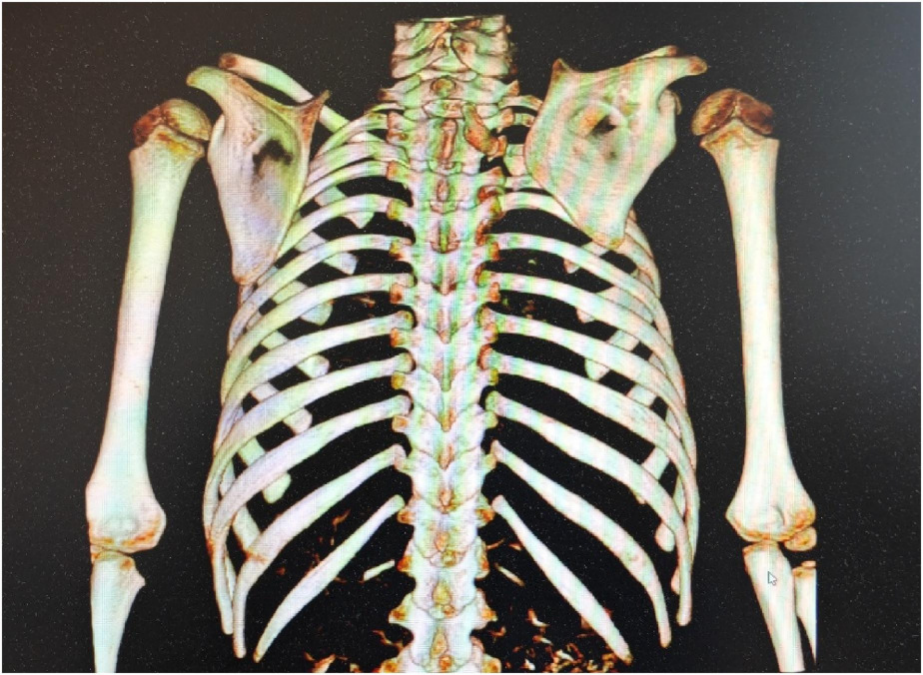
Case 11. Both the scapular and vertebral attachments of the omovertebral bone were cartilaginous.

Two distinct connection patterns were observed at the vertebral and scapular ends of the omovertebral bone. In one type, only the scapular attachment to the infraspinous portion was cartilaginous (Figure 8). In the other, both the scapular attachment to the infraspinous portion and the vertebral attachment were cartilaginous (Figure 9).

The mean height-to-width ratio of the affected side was 1.22, compared to the contralateral side (1.34), which demonstrated a significant decrease (Table 1). Compared to the contralateral side, the height of the affected scapula was decreased, and the width was increased. Only the difference in width reached statistical significance (Table 1). The mean superior displacement of the affected scapulae was 27.74 mm, with a mean rotational difference of 20.69°.

**Table 1:** The measurement of the scapula.

Parameters	Affected Side	Contralateral Side	*t*-statistic	*p*-value
	(Mean ± SD)	(Mean ± SD)		
Height (mm)	72.85 ± 5.84	75.97 ± 6.39	−0.70	0.499
Width (mm)	59.69 ± 6.83	56.38 ± 4.60	−3.58	**0** **.** **004**
Height-Width				
Ratio	1.22 ± 0.13	1.34 ± 0.08	2.27	**0** **.** **044**
95% CI	(1.126, 1.300)	(1.280, 1.404)		

The bold values (*p*-value < 0.05) means statistically significant.

**Figure 10:**
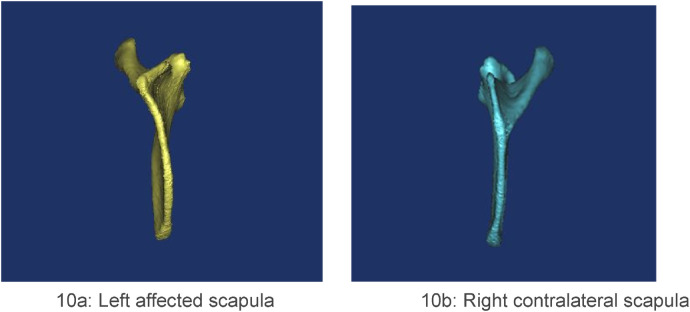
**(a,b)** case 12. The scapula was observed from the sagittal view. A characteristic anterior convexity was observed in the infraspinous portion of the affected scapula.

**Figure 11:**
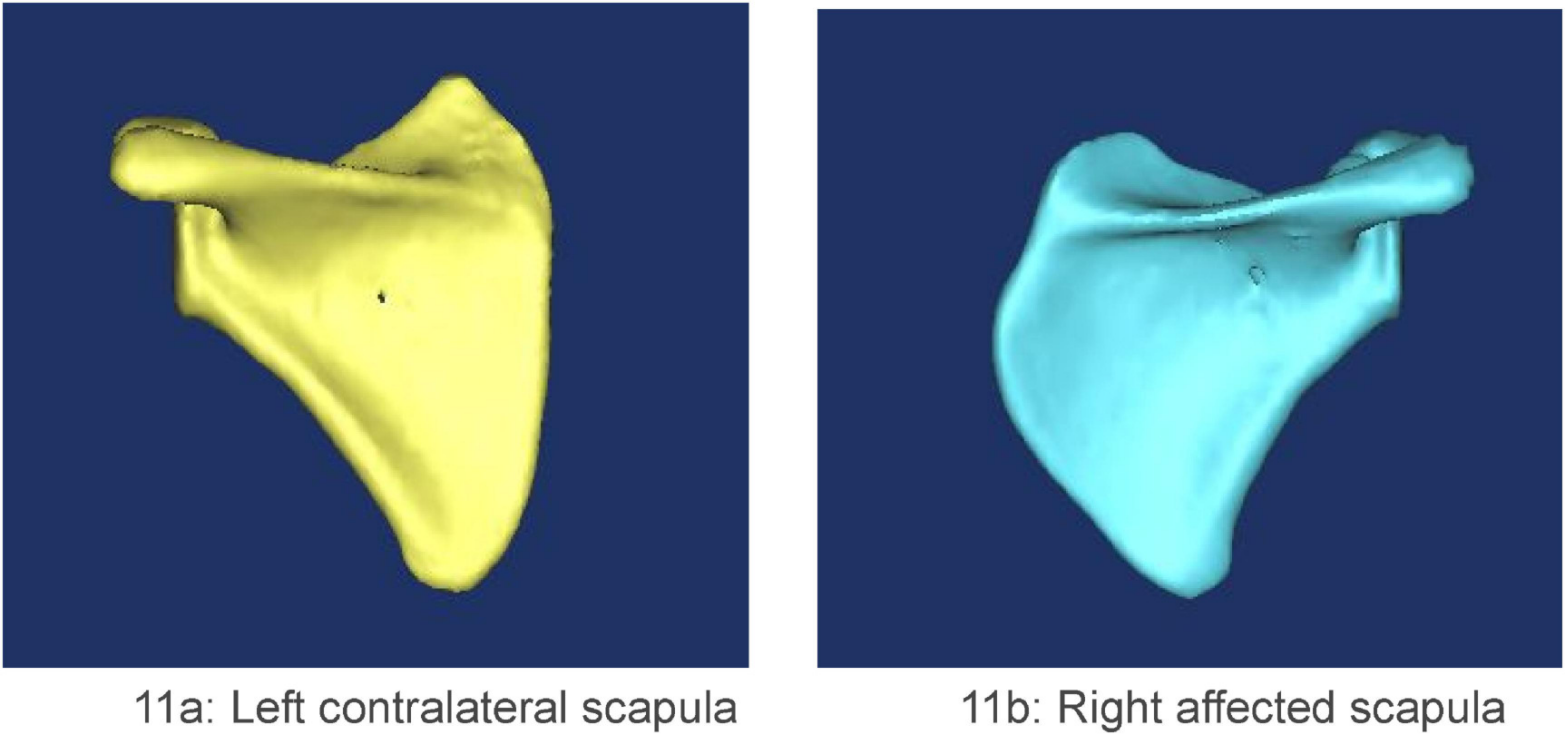
**(a, b)** case 1. The scapula was observed from the posterior view. The supraspinous portion of the vertebral border of the affected scapula displayed a predominantly convex contour.

The infraspinous portion of the affected scapula consistently exhibited an anterior curvature (Figures 10a,b). In contrast, the supraspinous portion of the vertebral border presented two morphologies: a convex contour predominated (Figures 11a,b), with only a single case displaying a concave border (Figure 1).

The scapula rotational angle and the omovertebral bone trunk angle were measured (Table 2). The Pearson correlation coefficient was −0.22, indicating no significant correlation between the two parameters.

**Table 2:** The angle of the scapula and omovertebral bone.

Parameters	Mean ± SD	*r*	*p*
Scapula rotational angle	19.85° ± 7.14°		
Omovertebral bone			
trunk angle	46.19° ± 26.42°		
		−0.22	>0.05

*r* = Pearson correlation coefficient.

All elevated scapulae exhibited characteristic morphologic changes: the vertebral border was conspicuously prominent, whereas the lateral border showed variable degrees of depression. One scapula also presented a widened and curved lateral edge at the inferior angle. The glenoid cavity retained its normal shape in most cases; only two demonstrated severe tilting. Rotational deformity was universal among the elevated scapulae.

Associated anomalies were present in all cases. Spina bifida was universal, while spinal fusion was identified in six cases. The remainder of the cohort exhibited either hemivertebrae or scoliosis, each found in two cases. Notably, no rib deformities were observed.

## Discussion

Given the rarity of Sprengel deformity, our cohort of 12 patients is consistent with previous two-center studies. However, the small sample size limits the generalizability of our findings, and statistical analyses should be interpreted with caution.

The etiology of Sprengel deformity remains elusive, though it is frequently associated with concomitant congenital malformations, as commonly observed in Klippel-Feil syndrome (15, 16). The deformity is attributed to an arrested embryonic development during the fifth to eighth gestational week, which fails caudal scapular migration.

The pathophysiology of this condition involves intricate pathological changes. The failure of descent is not merely a positional anomaly but is accompanied by a spectrum of morphological and structural alterations. The affected scapula is invariably hypoplastic, often with a characteristic convex superior border. The most significant pathological feature is the omovertebral bone, a fibro-cartilaginous or bony connection that tethers the superior vertebral border of the scapula to the spinous process, lamina, or transverse process of the cervical vertebrae (typically C4–C7). This abnormal structure is a primary cause of the scapula's fixation and restricted mobility (17, 18). Furthermore, the muscular anatomy is profoundly disturbed. The scapular muscles, particularly the trapezius, rhomboids, and levator scapulae, are hypoplastic, fibrotic, or partially replaced by fibrous bands, further contributing to the scapular fixation and functional deficit.

The mean height-to-width ratio in our cohort (1.22) was significantly lower than the normative value of 1.49 reported by Ogden et al. (19), confirming the dysplastic nature of the affected scapulae. The reduction is attributable to increased width at the convex vertebral border and decreased height secondary to anterior curvature of the supraspinous portion. This value falls within the range (0.94–1.60) reported by Cho et al. in their series of Sprengel deformity patients (14). The convexity always coincided with the omovertebral attachment site and is best explained by a chronic traction force on the medial border epiphysis. During normal development, the scapula descends. The omovertebral band restrains this descent and exerts continuous traction along its own axis, stimulating overgrowth of the vertebral border and producing the characteristic convexity. One scapula in our cohort exhibited a concave vertebral border at the bone's attachment; the mechanism is unclear but may reflect asynchronous growth between the epiphysis and the omovertebral bone, with the latter outpacing the former. Why this imbalance occurs remains unknown.

Cho et al. reported a mean rotational difference of 27° (range 5–53°) in unilateral Sprengel deformity (14); our cohort yielded a comparable 20.7° (13–36°). Wada et al. described a single left-sided case in which an omovertebral bone with a cartilaginous attachment to the infraspinous fossa was accompanied by minimal scapular rotation (20). In contrast, every scapula in the present series exhibited some rotational malposition, underscoring the wide morphologic and positional spectrum of the deformity.

The omovertebral tether is the critical determinant of scapular position. Earlier series have described three histologic variants—cartilaginous, fibrous, and osseous (21, 22)—with a reported prevalence ranging from 19% to 87% (12, 20). Willett and Walsham first documented the omovertebral bone in 1883 (23); Cavendish found it in only 19 of 100 patients (19%) in 1,972 (12), whereas Wada et al. identified it in 20 of 22 shoulders (87%) (20). Our incidence of 40% (12 of 30) falls between these previously reported extremes, highlighting the variable expressivity of this anomaly.

We assessed the morphological changes of the omovertebral bone from four perspectives: posterior, anteroposterior, sagittal, and coronal, following 3D-CT reconstruction. The reconstructed 3D-CT images clearly demonstrated the spatial location of the omovertebral bone. The majority of omovertebral bones tethered the convexity of the infraspinous segment of the medial scapular border; three, however, anchored to the convexity of the mesoscapular segment in this study. Carson depicted the connection as a rhomboid or trapezoid plate of mixed cartilage and bone (22); we encountered considerably more variability. Four bones were cylindrical, three were rhomboid, and five were irregular shapes. No correlation was demonstrable between the axis of the omovertebral bone and the magnitude of scapular rotation (the Pearson correlation coefficient −0.22, *P* > 0.05, Table 2).

The application of 3D-CT reconstruction in evaluating Sprengel deformity offers distinct and decisive advantages over conventional two-dimensional radiographs. Unlike plain radiographs, which provide limited, superimposed views, 3D-CT generates a clear, three-dimensional representation of the deformity. It accurately depicts the complex morphology of the omovertebral bone, its precise attachments to the scapula and spine, and the spatial relationships between these structures. Furthermore, 3D-CT is key for assessing the associated 3D scapular deformity—namely, its elevation, rotation, and the characteristic hypoplasia and curvature along the vertebral border. This comprehensive spatial analysis is vital for surgical planning, allowing surgeons to locate the tethering structure, gauge the degree of scapular distortion, and tailor the release and correction procedures. Consequently, 3D-CT moves beyond basic diagnosis to become an indispensable tool for detailed pathoanatomic evaluation, forming a solid basis for surgical intervention.

The findings of this study should be considered in light of its limitations. One is that the number of cases is small compared with some large series (12, 24). Another limitation is the absence of Magnetic Resonance Imaging. While MRI could better visualize cartilaginous structures and neural anomalies, it was not routinely performed due to the need for sedation in young children and the superior bony detail provided by CT. We acknowledge the radiation exposure of CT and advocate for judicious use with dose-reduction protocols. The unossified omovertebral bone cannot be noticed. And some concomitant anomalies, such as syringomyelia or tethered cord syndrome (24), cannot be recognized either. It would be valuable to include more cases and complement them with other techniques to better characterize the morphological spectrum of Sprengel deformity in children.

## Conclusion

By precisely delineating the diverse morphology and spatial location of the omovertebral bone and the complex scapular distortion in three dimensions, 3D-CT provides invaluable pathoanatomic data that significantly advances our understanding of Sprengel deformity in children.

## Data Availability

The original contributions presented in the study are included in the article/Supplementary Material, further inquiries can be directed to the corresponding author.
